# Has Pollination Mode Shaped the Evolution of *Ficus* Pollen?

**DOI:** 10.1371/journal.pone.0086231

**Published:** 2014-01-23

**Authors:** Gang Wang, Jin Chen, Zong-Bo Li, Feng-Ping Zhang, Da-Rong Yang

**Affiliations:** 1 Key Laboratory of Tropical Forest Ecology, Xishuangbanna Tropical Botanical Garden, Chinese Academy of Sciences, Mengla, Yunnan, China; 2 University of Chinese Academy of Sciences, Beijing, China; 3 Key Laboratory of Forest Disaster Warning and Control in Yunnan Province, College of Forestry, Southwest Forestry University, Kunming, China; 4 Key Laboratory of Economic Plants and Biotechnology, Kunming Institute of Botany, Chinese Academy of Sciences, Kunming, China; Montreal Botanical Garden, Canada

## Abstract

**Background:**

The extent to which co-evolutionary processes shape morphological traits is one of the most fascinating topics in evolutionary biology. Both passive and active pollination modes coexist in the fig tree (*Ficus*, Moraceae) and fig wasp (Agaonidae, Hymenoptera) mutualism. This classic obligate relationship that is about 75 million years old provides an ideal system to consider the role of pollination mode shifts on pollen evolution.

**Methods and Main Findings:**

Twenty-five fig species, which cover all six *Ficus* subgenera, and are native to the Xishuangbanna region of southwest China, were used to investigate pollen morphology with scanning electron microscope (SEM). Pollination mode was identified by the Anther/Ovule ratio in each species. Phylogenetic free regression and a correlated evolution test between binary traits were conducted based on a strong phylogenetic tree. Seventeen of the 25 fig species were actively pollinated and eight species were passively pollinated. Three pollen shape types and three kinds of exine ornamentation were recognized among these species. Pollen grains with ellipsoid shape and rugulate ornamentation were dominant. Ellipsoid pollen occurred in all 17 species of actively pollinated figs, while for the passively pollinated species, two obtuse end shapes were identified: cylinder and sphere shapes were identified in six of the eight species. All passively pollinated figs presented rugulate ornamentation, while for actively pollinated species, the smoother types - psilate and granulate-rugulate ornamentations - accounted for just five and two among the 17 species, respectively. The relationship between pollen shape and pollination mode was shown by both the phylogenetic free regression and the correlated evolution tests.

**Conclusions:**

Three pollen shape and ornamentation types were found in *Ficus*, which show characteristics related to passive or active pollination mode. Thus, the pollen shape is very likely shaped by pollination mode in this unique obligate mutualism.

## Introduction

Pollen is one of the key reproductive characters of flowering plants, and has been directly selected upon in the coevolutionary history of plant and pollination vectors [1], [2]. Pollen morphology is considered as a phylogenetic conserved trait and has been used in systematic studies in some families, such as Acanthaceae [3], Moraceae [4]–[6], Scrophulariaceae [7] and Orchidaceae [8], [9]. Many other studies suggest a strong correlation between pollen characters and pollination mode [10], [11]. Psilate (smooth) pollen is often associated with abiotic pollination (wind or water), and elaborate pollen ornamentation is correlated with biotic pollination, especially entomophily [12]–[14]. Whether pollination modes influence pollen evolution is controversial, but is arguably best considered using plant groups that have high species diversity and robust phylogenic relationships, are at a lower taxonomic level (genus or lower), and have a long history of different pollination modes coexisting.

The fig tree and pollinating fig wasp association is a classic example of a coevolved mutualism [15]. In their mutual dependency for successful reproduction, fig and fig wasp show sophisticated adaptations to each other in both morphology and phenology [16]. Fig trees, including over 750 species, are defined by the enclosed inflorescence (syconium) with a narrow bract-lined ostiole. Fig wasps, the only pollinator of fig trees, gain access to the syconium through the ostiole, using their mandibular appendages and strong legs. Wasps pollinate the flowers, and oviposit inside the ovules between the integument and the nucellus, introducing their ovipositors through the style. An egg is only deposited if the ovipositor is long enough to reach that location [17], [18].

Two pollination modes, active and passive pollination, coexist across the fig and fig wasp mutualism. Based on the systematic survey of Kjellberg *et al.* [19], almost two thirds of fig species are actively pollinated and the others are passively pollinated. Less pollen is produced in actively pollinated figs, in which fig wasps actively collect pollen from anthers, store them in special pollen pockets and deposit pollen efficiently on the stigmas within other receptive syconia using their forelegs [20]. In other fig wasps, the above behavior is absent and there are often neither coxal combs nor pollen pockets on their body. Passively pollinated figs usually produce large quantities of pollen and wasps are coated with pollen as they emerge from the inflorescence [21]. Molecular studies estimate that the fig-fig wasp mutualism has been established for about 75 Myr [15], while fossil records of fig wasps show that active pollination has existed for at least 34 Myr [22]. Passive pollination has been inferred as the ancestral mode in this mutualism, followed by single or multiple origins of active pollination and several independent losses [23], [24], while a recent study suggested that ancestral pollination modes are equivocal with independent multi-shifts between passive and active states [15].

High species diversity, the co-occurrence of two pollination modes and a long coevolutionary history with its obligate pollinator makes *Ficus* ideal for inferring the role of pollination mode shifts on pollen evolution. However, until now the pollen of only 45 fig species has been described, in scattered reports focusing on different pollen flora or pollen rain investigations [25], [26]. Systematic studies of the pollen morphology in the genus are still insufficient, except for recent study of pollen observations for 28 fig species in Taiwan conducted using SEM [4]. No research has yet focused on the relationship between pollen morphology and the pollination mode of *Ficus*. In this study, by investigating pollen morphology and pollination mode of twenty-five *Ficus* species in a northern region of tropical Asia, we aimed to understand how pollination mode shifts influence the evolution of *Ficus* pollen morphology.

## Materials and Methods

### Plant sampling and site

**Figure 1:**
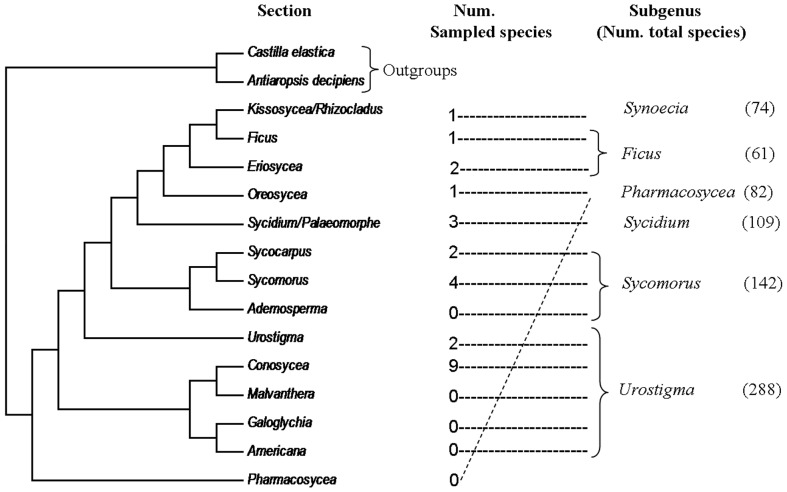
Distribution of sampled species in a section level modified *Ficus* tree by Cruaud et al. (2012). Number of sampled species in this study from each section and total species number of each subgenus were labelled.

Pollen samples of 25 *Ficus* species were collected from living trees in or close to Xishuangbana Tropical Botanical Garden (21°41′N, 101°25′E), Yunnan, China. The sample, though small, is representative of the *Ficus* phylogeny: the samples represent all six subgenera of *Ficus* and 10 of 19 sections of the morphological taxonomy [25] (Table S1), and 9 of 14 phylogenetic clades from the most current *Ficus* phylogeny [15] (Figure 1). One to seven species were included in each phylogenetic clade. Eleven out of 25 sampled species belong to subgenus *Urostigma* (Figure 1), which covers about one-third of the approximately 800 global *Ficus* species [25]. Though all sampled species were collected from one geographical area, most of the species are widely distributed in tropical Asia, the home for almost half of global *Ficus* species [25]. Pollen sampled from fresh figs are used in this study (for reasons explained below) hence limiting our sample size. Considering the low diversity of *Ficus* pollen [4], [25], 25 sampled species with two pollination modes should be adequate for a descriptive study on the influence of pollination mode change on pollen morphology evolution. To establish the phylogenetic relationships of the 25 fig species, leaf samples from two species, *F. esquiroliana* and *F. sarmentosa* var. *henryi*, were collected from the same location (for DNA sequencing), and sequences of the other 23 *Ficus* species, and two outgroup species (*Antiaropsis decipiens* and *Castilla elastica*), were included from GenBank (Table S1). The study was approved by the Xishuangbanna Tropical Botanical Garden, Chinese Academy of Science, which is the owner of the site.

### Pollination mode identification

As pollen grains of *Ficus* are often very small (7–22 µm) and difficult to count, the anther/ovule ratio (A/O ratio), instead of pollen/ovule ratio, has often been used in pollination mode identification [19], [23]. Based on previous systematic survey results of Kjellberg et al. [19] active pollination was inferred when the A/O ratio was below 0.16 and passive pollination was inferred for A/O ratios larger than 0.21. Almost no reported species has an A/O ratio lying between 0.16 and 0.21, which suggests a strong selection pressure from the pollination behavior of fig wasps. The A/O ratio of each species was measured by counting numbers of anthers and female flowers in 3 to 67 figs from three trees in most cases. Examining one fig from a single tree is sufficient to establish the pollination mode [19].

### Pollen observation with SEM

More than 20 mature stamens from two to four trees from each species were collected from fresh figs and acetolyzed using a modified version of Erdtman's method [27]. Pollen grains were coated with gold, and observed under an SEM (S-4800) to measure pollen size (20 grains per species), pollen shape and detail exine ornamentations under 2000×, 7000× and 20000×, respectively. Pollen grains from dry herbaria specimens were not used here, for two reasons. First, because the pollen exine is very thin (∼1 µm), pollen grains from herbaria easily deform or break in the SEM environment [4]. Also, few mature pollen can be found inside syconia on herbaria specimens of *Ficus*, as the mature pollen is only available in the male flower phase when figs are nearly mature and falling from the branch, a stage in which they may not be frequently collected. Unfortunately, we were further limited in our sampling from living trees, because the low population density and asynchronous phenology of most *Ficus* species [25] increases the difficulty of getting stamens from many fresh figs in male flower phase. All these factors contribute to limit our sample size and also perhaps to explain the relatively little pollen research that has yet to be conducted in *Ficus* [4].

### Phylogenetic tree and trait mapping

Two nuclear genes (ITS and G3pdh) of all 25 fig species and two outgroup taxa from tribe *Castilleae* were used to establish a robust phylogenetic tree (Table S1). Extraction, PCR and sequencing methods for *F. esquiroliana* and *F. sarmentosa* var. *henryi* followed the methods of Xu et al. [28], and sequence data for the other 25 species (including the two outgroup taxa) were taken from GenBank (Table S1). Sequences were aligned with MEGA 5.0 [29]. The partitioned ITS+G3pdh dataset Bayesian Inference analysis was run for 50 million generations for three times using Mrbayes 3.1.1 [30] with GTR+G (ITS) and HKY+G (G3pdh) models selected as the best fit model by hierarchical likelihood ratio tests in jModeltest 0.1.1 [31]. Here, 50 million generations in the Bayesian analysis is acceptable, as the same topology was obtained in three simulations, and the average standard deviation of split frequencies reached 0.0007 to 0.0006 after 30 million generations, suggesting a very good indication of convergence according to the Mrbayes manual [30]. Pollination mode, pollen shape and exine ornamentation types were mapped on the phylogenetic tree of figs with the “ape” package [32] of R [33].

### Data analysis

The influence of pollination mode, A/O ration and sex system on two continual pollen traits, equatorial axis length (E) and polar axis/equatorial axis value (P/E value), was analysed with phylogenetic free regression, which was performed by controlling phylogenetic auto-correlation using Moran's eigenvectors as covariates with function ‘me.phylo’ in ‘adephylo’ package [34] of R.

To analyse the correlated evolution between pollen discrete traits (pollen shape and exine ornamentation types) and pollination mode and sex system of figs, the BayesTraits program [35] was used. The BayesDiscrete test module was used to investigate correlated evolution between pairs of discrete binary traits by comparing the fit (log likelihood) of two continuous-time Markov models. The first model assumes that the two traits (e.g., pollen shape type and pollination mode) evolve independently on the tree, whereas the second model allows two traits evolve in a correlated process such that the change rate in one trait depends on the background state of the other. The correlated pattern was determined by the Log Bayes factor (Log BF), which was calculated by likelihood for independent mode (L(I)) and dependent mode (L(D)), with a formula: Log BF = −2[L(D)−L(I)]. Log BF below 2 means weak evidence for correlated evolution, above 2 indicates positive evidence, 5–10 means strong evidence, and above 10 suggests very strong evidence [35]. Pollen shape and ornamentation types were recoded as binary characters to meet the requirement of the BayesDiscrete test.

## Results

### Pollen traits and pollination mode of figs

**Figure 2:**
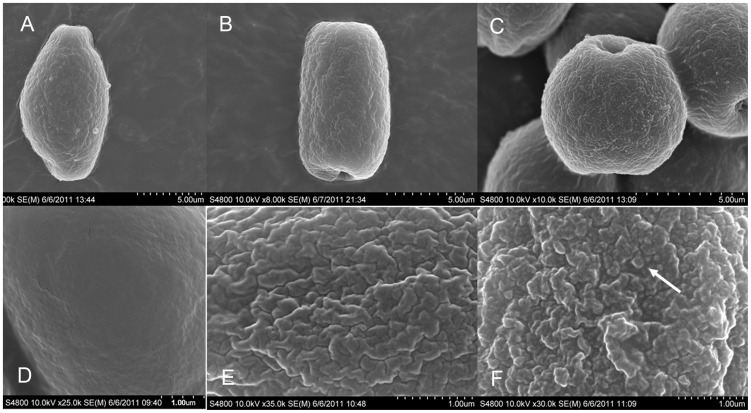
Pollen shape (a–c) and exine ornamentation types (d–f) of figs under a scanning electron microscope. (a) ellipsoid, *Ficus maclellandii*; (b) cylinder, *F. ischnopda*; (c) sphere, *F. langkokensis*, (d) psilate, *F. hispida*; (e) rugulate, *F. annulata*; (f) granulate-rugulate, *F. callosa*, the arrow indicate granule.

Consistent with previous studies, *Ficus* pollen grains are radially symmetrical, pollen size ranges from minutae to perminutae with polar axis (P): 6.64 (4.13–9.02) µm, equatorial axis (E): 9.87 (7.29–14.20) µm, and P/E value: 0.69 (0.45–0.96). Based mainly on the shape of pollen end [36], three pollen shapes were found in equatorial view: elliptical, rectangular and circular, corresponding to ellipsoid, cylinder and sphere in three-dimensional view. Ellipsoid pollen has an acute end, while cylindrical and sphere shaped pollens have obtuse ends (Figure 2). All species have pollen grains with 2-porate and some species have minor percentages of 3-porate pollen grains (Table S1). Three exine ornamentation types were found: psilate ornamentation (smooth) and two scabrate types: rugulate and granulate-rugulate ornamentation [37]. Ellipsoid shape (Figure 2, a) and rugulate ornamentation (Figure 2, e) are dominant types. Based on A/O ratio values (Table S1), 17 of the fig species are actively pollinated, and eight species are passively pollinated. More pollen traits and detailed information of the 25 fig species are presented in Table S1.

### Ficus phylogeny and traits mapping

**Figure 3:**
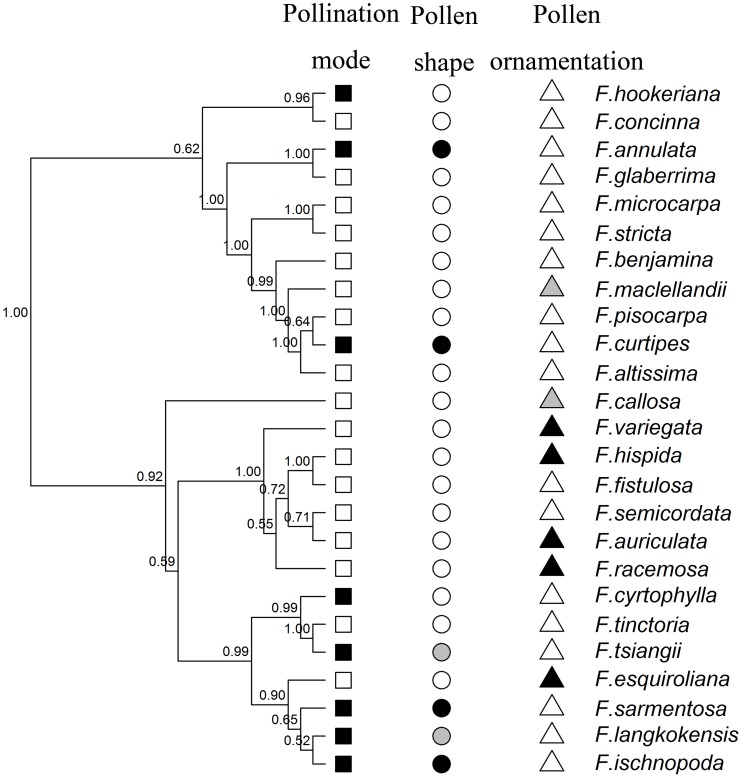
Bayesian tree of 25 fig species with pollination mode and pollen traits of figs. Posterior probability values are listed above the branches. Pollination mode (Square, black = passive pollination, white = active pollination); Pollen shape (Circle, black = cylinder, grey = sphere, white = ellipsoid) and (C) Pollen exine ornamentation (Triangle, black = psilate, grey = granulate-rugulate, white = rugulate). Two pollination modes, three pollen shapes and three kinds of exine ornamentation all appear to be examples of convergent evolution. Psilate and granulate-rugulate ornamentation only occur in active pollinated figs, while all cylinder and sphere pollens belong to passive pollinated figs.

There was strong support (posterior probability above 0.9) at most nodes in the fig phylogenetic tree, which is congruent with previous studies in most main clades (in section levels) (Figure 1) [15], [28]. However, the location of the section *Urostigma* (*F. concinna*, *F. hookeriana*) and section *Oreosycea* (*F. callosa*) and the relationships of those deeper nodes are not strongly supported in recent global *Ficus* phylogeny [15]. The two pollination modes, three pollen shapes and three exine ornamentation types of figs appear to be convergent traits as they all exist in different phylogenetic clades as shown in Figure 3.

### Relationship between pollen types and pollination modes

Ellipsoid pollen occurred in all 17 species of the actively pollinated fig species, while for the eight passively pollinated species, two had ellipsoid pollen, four species had cylindrical pollen, and two species had spherical pollen. On the other hand, all eight passively pollinated figs and 10 of the 17 actively pollinated figs presented rugulate ornamentation, while the other kinds (psilate and granulate-rugulate) only occurred in actively pollinated species (five and two species respectively) (Table 1). Significant correlations were found between P/E value and A/O ratio as well as P/E value and pollination mode in phylogenetic free regression (Table 2). The BayesDiscrete test showed strong correlated evolution only between pollen shape and pollination mode (Log BF = 7.47), positive correlated evolution between the pollen ornamentation and sex system (Log BF = 2.47), and weak correlated evolution in other traits pairs such as pollen ornamentation and pollination mode, pollen shape and sex system, pollen shape and pollen ornamentation as well as pollination mode and sex system (Table 3).

**Table 1:** Allocation of pollen shape and exine ornamentation types between two pollination modes.

Pollination mode	Pollen shape			Exine ornamentation		
(total *Ficus* spp.)	ellipsoid	cylinder	sphere	psilate	rugulate	granulate-rugulate
Active (17)	17	0	0	5	10	2
Passive (8)	2	4	2	0	8	0
Total (25)	19	4	2	5	18	2

**Table 2:** Analysis results of continual pollen traits with phylogenetic free regression.

Response	Predictor	Estimate	SE	*t*-value	*P*
Equatorial axis length	A/O ratio	−3.781	3.116	−1.213	0.240
	Pollination mode	−0.919	1.831	−0.502	0.622
	Sex system	−1.227	1.445	−0.847	0.408
P/E value	**A/O ratio**	0.607	0.187	3.248	**0.004**
	**Pollination mode**	0.302	0.110	2.749	**0.013**
	Sex system	−0.020	0.087	−0.229	0.822

**Table 3:** BayesDiscrete test results between binary pollen traits, pollination mode and sex system using the BayesTratis program for 25 *Ficus* species.

Traits correlation	Independent*	Dependent*	Log Bayes factor
**Pollen shape VS Pollination mode**	−31.559	−27.856	**7.407**
Pollen ornamentation VS Pollination mode	−29.219	−28.998	0.441
Pollen shape VS Sex system	−25.114	−25.330	−0.430
**Pollen ornamentation VS Sex system**	−22.296	−21.060	**2.471**
Pollen shape VS Pollen ornamentation	−28.282	−28.293	−0.022
Pollination mode VS Sex system	−26.095	−26.019	0.151

* Probabilities calculated as Log(harmonic mean);

Log Bayes factor: <2 weak evidence, >2 positive evidence, 5–10 strong evidence, >10 very strong evidence; Bold words mean terms with strong or positive evidence from both tests.Pollen shape and ornamentation types were recoded as binary characters as follows: Pollen shape: pollen with obtuse end (ellipsoid): ‘0’, pollen with acute end (cylinder and sphere): ‘1’; Pollen ornamentation: scabrate (rugulate and granulate-rugulate): ‘0’, smooth (psilate): ‘1’.

## Discussion

The 75 Myr fig-fig wasp mutualism provides a model system for targeting the role of pollination mode shifts on pollen evolution. Previous research suggested that there is little infrageneric variation in *Ficus* pollen [4], [25]. However, even in the small sample size of 25 fig species, we found three pollen shapes and three kinds of exine ornamentation. Ellipsoid pollen shape and rugulate ornamentation are the dominant characteristics. Pollen grains of actively pollinated figs are all ellipsoid in shape and cover three ornamentation types, while pollen grains from passively pollinated figs are all rugulate ornamentation and cover three shape types with six of eight species have obtuse end pollen (four cylinder and two sphere). Phylogenetic free regression showed the P/E value (another measurement of pollen shape) was significantly correlated with both pollination mode and A/O ratio. That was reinforced in the BayesDiscrete test that showed strong correlated evolution between pollen shape and pollination mode. These results suggest a high degree of convergent evolution of pollen shape, rather than phylogenetic conservatism, has influenced pollen shape in *Ficus*.

Both pollen shape and exine ornamentation types correspond with adaptation to different pollination modes. Ellipsoidal pollen, which has an acute end, occurs in all actively pollinated figs, while cylindrical and spherical pollens, which have obtuse ends, are only found in passively pollinated figs. Selection may have favored a larger surface area per unit volume for pollen of passively pollinated figs (cylindrical and spherical vs. ellipsoidal) as they have a higher probability of adhering to wasp bodies and being transported. Significant correlation between P/E value and pollination mode (and A/O ratio), and strong correlated evolution between pollen shape and pollination mode also support this conclusion. Although there is weak correlation between exine ornamentation and pollination mode, just as in other common entomophilous plants, it is possible that rugulate ornamentation, which adheres well to the body of the pollinator, is more favorable in passively pollinated figs [14]. Contrarily, rugulate ornamentation may contribute little to pollen collection for active fig wasps that possess highly sophisticated behavioral and morphological traits (such as pollen pockets and coxal combs) to collect and store pollen efficiently [18], [19]. This may also explain why rugulate ornamentation was found in all passively pollinated figs, and psilate ornamentation was only found in actively pollinated figs. Adaptation to different pollination modes of fig wasps may be the main driving factor on the divergence of pollen shape. Considering the weak correlation between pollen ornamentation and pollination mode, rechecking the role of pollination mode shifts on ornamentation evolution with a greater sample size is needed in further studies.

Absence of evolutionary correlation between exine ornamentation and pollination mode, and ellipsoid pollen in two passively pollinated figs might be attributed to several competing but not necessarily mutually exclusive hypothesis. First, trait evolution inertia may explain this result, as psilate pollen only occurs in figs with the lowest A/O ratio, which may correspond to extreme active pollination. This idea is supported by the significant correlation found between the P/E value and A/O ratio. Similar psilate and ellipsoid fossil pollens found in the pollen pocket of a 34 Myr old fossil fig wasp [22] also demonstrates the stability of pollen morphology. Second, it is likely that there have been multiple shifts in pollination mode throughout evolutionary history confounding the direction and process of pollen evolution. Even the driving factor of pollinator mode shifts is still unclear, as it is possible that multiple shifts between active and passive pollination mode would reverse the selection direction of pollination mode on pollen evolution [15], [23]. Third, it is also possible that hybridization between figs from different pollination modes [38] have contributed to the mixed patten of pollen morphology evolution. Cyto-nuclear discordance checking among closely related species with different pollination modes may supply evidence on this point in future [39]. Fourth, some of our results may be due to our sample size limitations. If, for example, we were to find psilate ornamentation in more clades, it might change the relationship between pollen ornamentation and pollination mode.

The positive correlated evolution between pollen ornamentation and sex system in BayesDiscrete test is a particularly interesting finding (Table 3), and the driving factor behind the relationship should be the subject of further research. The relationship may be unstable and require rechecking with a greater sample size, as the Log BF value (2.471) is just a little higher than 2 (the low edge of positive evidence). In contrast, the correlated evolution between pollen shape and pollination mode is robust, as shown by both the high Log BF value (7.407) in BayesDiscrete test and the significant correlation (*P* = 0.013) in phylogenetic free regression.

Of the 40 recognized Moreacea genera, *Ficus* is the only genus which has both active and passive pollination mode by highly obligate insect pollinators [25], [40]. *Ficus* pollens are smaller in size and have smoother exines than pollens of other Moraceae genera, which are larger (equatorial axis: 10–40 µm), have scabrate or granular ornamentation, are spheroidal in shape and pollinated by wind or common insects [5], [6], [25], [41]. *Ficus* is the only Moreacea genus with psilate pollen grains, and the smaller pollen of *Ficus* may be an adaptation to the small body size of its obligate pollinator. Systematic pollen morphological comparation with more Moreceae genera, which have different pollination modes and clear phylogenetic history are needed to understand whether the fig/fig wasp mutualism has exclusively shaped the traits of *Ficus* pollen, especially for the sister group of *Ficus*, tribe *Castilleae* (e.g. genus *Antiaropsis*, *Castilla*) [40], [42], which was found to be pollinated by another small pollinator, thrips, and may also have obligate mutualistic relationships with pollinators [43], [44]. The obligate pollination relationship in tribe *Castilleae* may not be as exclusive as that in the *Ficus* genus, as thrips are pollinators of many plant families [44]. Unfortunately, knowledge on the pollen morphology and evolutionary history of *Castilleae*-thrips system are still scanty. Comparative researches including Tribe *Castilleae* will improve our understanding of pollen evolution when pollination mode shifts from common entomophily to obligate pollination.

Correlations between pollen morpology and pollination mode have also been reported in many other systems. In the subfamily Papilionoideae (Leguminosae), pollen from bird or bat- pollinated species is coarsely rugulate or verrucate, while insect pollinated plants have pollen with simple reticulate or perforate surface sculpturing [45]. Within the genus *Erythrina* (Leguminosae), plants pollinated by hummingbirds and passerine birds differ in granule density and lumina size of their pollen [46]. With the exception of five fig species in our study, entomophilous psilate pollen has mainly been reported from beetle pollinated Araceae, while pollens with elaborate ornamentations occur in other non-beetle pollinated Araceae [11]. Within the genus *Harpalyce* (Leguminosae), pollen of bird-pollinated species is stickier than that of insect-pollinated species and has a higher level of pollenkitt [47]; it is still unclear whether pollen from passively pollinated figs is stickier than actively pollinated figs. Together, our results and those of the studies mentioned above suggest pollination mode shifts increase the diversity of pollen characteristics.

In conclusion, with 25 species from most main phylogenetic clades, we found a relative high diversification of *Ficus* pollen morphology: three pollen shapes and three pollen ornamentation types, which suggest adaptation in some degree to passive or active pollination mode. Pollen shape and pollination mode were demonstrated to be evolutionary correlated. It is very likely pollen shape has been shaped by the shifts in pollination mode in the fig-fig wasp mutualism system. The study provides a foundation for our understanding of how the long coevolutionary history between plants and obligate pollinators influences pollen evolution. A logical next step for research is observation on pollen and pollination mode identification of more species from the *Ficus* genus and other Moraceae genera, especially from tribe *Castillaea*.

## Supporting Information

Table S1 **Pollen characteristics of** ***Ficus*** **and outgroup species included in the present study.** Anther/ovule ratio, pollination mode and GenBank accession numbers are also provided. Sequences produced for this study are indicated in bold font.
(DOC)
