# Supplementary material for: Has Pollination Mode Shaped the Evolution of Ficus Pollen?
Source: PLoS One. 2014 Jan 23;9(1):e86231. doi: 10.1371/journal.pone.0086231 (PMC3900511; doi:10.1371/journal.pone.0086231)
Supplement: Table S1 — Pollen characteristics of Ficus and outgroup species included in the present study. Anther/ovule ratio, pollination mode and GenBank accession numbers are also provided. Sequences produced for this study are indicated in bold font. (DOC) [file pone.0086231.s001.doc]

**Table S1. Pollen characteristics of *Ficus* and outgroup species included in the present study.** Anther/ovule ratio, pollination mode and GenBank accession numbers are also provided. Sequences produced for this study are indicated in bold font.

| **Species** | **Polar axis (P, μm)*** | **Equatorial axis (E, μm)*** | **P/E value*** | **Shape type** | **Ornamentation types** | **Numb.**  **aperture** | **Anther/ovule ratio** | **Pollination mode/Sex system** | **Accession numbers of ITS/G3pdh[1-4]** |
| --- | --- | --- | --- | --- | --- | --- | --- | --- | --- |
| **Subg. *Ficus* Sect. *Ficus*** | | | | | | | | | |
| *F.ischnopoda* | 5.87±0.54 | 9.38±0.64 | 0.63±0.08 | rectangular | rugulate | 2 | 0.36 ± 0.04（n=35） | Passive/Dioecious | AY730122/EF092380 |
| **Sect.** ***Eriosycea*** | | | | | | | | | |
| *F.esquiroliana* | 5.80±0.48 | 9.07±0.71 | 0.64±0.07 | elliptical | psilate | 2 | 0.04（n=3） | Active/Dioecious | JQ773872/**KF811032** |
| *F.langkokensis* | 6.00±0.44 | 7.29±0.42 | 0.83±0.07 | circular | rugulate | 2 | 0.68 ± 0.17（n=40） | Passive/Dioecious | JN117638/JN117703 |
| **Subg. *Synoecia* Sect. *Rhizocladus*** | | | | | | | | | |
| *F.sarmentosa* var. *henryi* | 5.01±0.51 | 11.28±0.96 | 0.45±0.06 | rectangular | rugulate | 2 | 0.4 ± 0.10（n=29） | Passive/Dioecious | EU087679 /**KF811033** |
| **Subg. *Syicidium* Sect. *Sycidium*** | | | | | | | | | |
| *F.tsiangii* | 8.12±0.39 | 8.50±0.43 | 0.96±0.04 | circular | rugulate | 2 | 0.94 ± 0.18（n=20） | Passive/Dioecious | JN117650/JN117714 |
| *F.cyrtophylla* | 6.37±0.48 | 7.47±0.63 | 0.86±0.10 | elliptical | rugulate | 2 | 0.49 ± 0.11 （n=20） | Passive/Dioecious | JN117628/JN117694 |
| **Sect. *Palaeomorphe*** | | | | | | | | | |
| *F.tinctoria* | 7.20±0.68 | 10.91±0.98 | 0.67±0.11 | elliptical | rugulate | 2 (3) @ | 0.05 ± 0.01（n=29） | Active/Dioecious | JN117649/JN117713 |
| **Subg. *Sycomorus* Sect. *Sycomorus*** | | | | | | | | | |
| *F.racemosa* | 6.75±0.57 | 11.51±2.40 | 0.61±0.12 | elliptical | psilate | 2 | 0.07^[5]^ | Active/Monecious | AF165405/JN126051 |
| *F.variegata* | 6.92±0.46 | 11.31±0.65 | 0.61±0.06 | elliptical | psilate | 2 (3) @ | 0.05^[5]^ | Active/Dioecious | AF165415/DQ367633 |
| *F.auriculata* | 6.90±0.88 | 11.06±1.32 | 0.63±0.07 | elliptical | psilate | 2 | 0.02 ± 0.01（n=4） | Active/Dioecious | AF165376/JN117685 |
| **Sect. *Hemicardia*** | | | | | | | | | |
| *F.semicordata* | 5.25±0.51 | 7.64±0.65 | 0.69±0.09 | elliptical | rugulate | 2 (3) @ | 0.03 ± 0.04（n=20） | Active/Dioecious | JN117646/JN117710 |
| **Sect. *Sycocarpus*** | | | | | | | | | |
| *F.fistulosa* | 4.13±0.26 | 7.61±0.47 | 0.54±0.05 | elliptical | rugulate | 2 | 0.02 ^[5]^ | Active/Dioecious | JN117629/JN117695 |
| *F.hispida* | 5.34±0.58 | 8.81±1.09 | 0.61±0.10 | elliptical | psilate | 2 | 0.03 ± 0.02（n=20） | Active/Dioecious | JN117634/JN117700 |
| **Subg. *Pharmacosycea* Sect. *Oreosycea*** | | | | | | | | | |
| *F.callosa* | 7.33±0.65 | 10.41±1.25 | 0.72±0.12 | elliptical | granulate-rugulate | 2 | 0.08 ^[5]^ | Active/Monecious | AY063565/EF092367 |
| **Subg. *Urostigma* Sect. *Urostigma* Subsect. *Conosycea*** | | | | | | | | | |
| *F.altissima* | 7.66±0.51 | 9.99±0.77 | 0.77±0.07 | elliptical | rugulate | 2 (3) @ | 0.09 ± 0.02 (n=30) | Active/Monecious | JN117617/EU087621 |
| *F.benjamina* | 7.15±0.57 | 12.74±0.82 | 0.56±0.07 | elliptical | rugulate | 2 (3) @ | 0.10 ± 0.02 (n=26) | Active/Monecious | JN117620/JN117687 |
| *F.stricta* | 7.37±0.87 | 9.17±0.77 | 0.81±0.09 | elliptical | rugulate | 2 | 0.15 ± 0.02 (n=29 | Active/Monecious | JN117647/JN117711 |
| *F.curtipes* | 5.53±0.52 | 7.95±0.95 | 0.70±0.06 | rectangular | rugulate | 2 (3) @ | 0.84 ± 0.10 (n=67) | Passive/Monecious | JN117627/JN117693 |
| *F.microcarpa* | 7.43±0.53 | 8.94±0.68 | 0.83±0.07 | elliptical | rugulate | 2 | 0.13^[5]^ | Active/Monecious | JN117640/JN117705 |
| *F.glaberrima* | 6.09±0.42 | 7.96±0.71 | 0.77±0.08 | elliptical | rugulate | 2 | 0.11 ± 0.02 (n=42) | Active/Monecious | JN117630/JN117696 |
| *F.maclellandii* | 7.86±0.64 | 11.19±1.03 | 0.71±0.11 | elliptical | granulate-rugulate | 2 | 0.14 ± 0.04 (n=34) | Active/Monecious | JN117639/JN117704 |
| *F.pisocarpa* | 9.02±1.89 | 14.20±3.74 | 0.65±0.09 | elliptical | rugulate | 2 | 0.13 ± 0.04（n=10） | Active/Monecious | JN117643/JN117707 |
| *F.annulata* | 5.07±0.50 | 10.64±0.83 | 0.48±0.06 | rectangular | rugulate | 2 | 0.38 ± 0.03(n=15) | Passive/Monecious | JN117618/EU087622 |
| **Sect. *Urostigma* Subsect. *Urostigma*** | | | | | | | | | |
| *F.concinna* | 6.82±0.49 | 10.25±0.76 | 0.67±0.06 | elliptical | rugulate | 2 | 0.13 ± 0.01 (n=40) | Active/Monecious | JN117626/JN117692 |
| *F.hookeriana* | 9.00±0.83 | 11.33±0.99 | 0.80±0.07 | elliptical | rugulate | 2 (3) @ | 0.56 ± 0.14 (n=16) | Passive/Monecious | JN117636/JN117702 |
| **Outgroups from Tribe *Castilleae*** | | | | | | | | | |
| *Antiaropsis decipiens* | |  |  |  |  |  |  |  | AY730142/EF092326 |
| *Castilla elastica* | |  |  |  |  |  |  |  | AY730143/EF092327 |

Notes: *, mean± s.d; @, few grains have three apertures with triangular shape in equatorial view; Sections identification here following classical taxonomy [6]. Two sections within Subgenus *Syicidium* were combined, Section *Hemicardia* was included within Section *Sycomorus*, and Subsection *Consycea* and *Urostigma* were taken as two independent sections in recent phylogenetic work [7].

**References:**

1. Ronsted N, Weiblen GD, Clement WL, Zerega NJC, Savolainen V (2008) Reconstructing the phylogeny of figs (*Ficus*, Moraceae) to reveal the history of the fig pollination mutualism. Symbiosis 45: 45-55.

2. Ronsted N, Weiblen GD, Cook JM, Salamin N, Machado CA, et al. (2005) 60 million years of co-divergence in the fig-wasp symbiosis. Proc R Soc Lond B Biol Sci 272: 2593-2599.

3. Li HQ, Chen JY, Wang S, Xiong SZ (2012) Evaluation of six candidate DNA barcoding loci in *Ficus* (Moraceae) of China. Mol Ecol Resour 12: 783-790.

4. Xu L, Harrison RD, Yang P, Yang D-R (2011) New insight into the phylogenetic and biogeographic history of genus *Ficus*: Vicariance played a relatively minor role compared with ecological opportunity and dispersal. J Syst Evol 49: 546-557.

5. Kjellberg F, Jousselin E, Bronstein JL, Patel A, Yokoyama J, et al. (2001) Pollination mode in fig wasps: the predictive power of correlated traits. Proc R Soc Lond B Biol Sci 268: 1113-1121.

6. Berg CC, Corner EJH (2005) Moraceae (*Ficus*). In: Nooteboom HP, editor. Flora Malesiana Series I -Seed Plants. Leiden: Nationaal Herbarium Nederland. pp. 50-51.

7. Cruaud A, Rønsted N, Chantarasuwan B, Chou LS, Clement WL, et al. (2012) An extreme case of plant-insect co-diversification: figs and fig-pollinating wasps. Syst Biol 61: 1029-1047.
